# The frequency and importance of chest pain in midterm follow up of transcatheter closure of interatrial septal defect

**DOI:** 10.15171/jcvtr.2017.37

**Published:** 2017-12-25

**Authors:** Azin Alizadehasl, Mohsen Neshati Pir Borj, Anita Sadeghpour, Ata Firouzi, Hamidreza Sanati, Masoud Movassaghi

**Affiliations:** ^1^Echocardiography Research Center, Rajaie Cardiovascular Medical & Research Center, Iran University of Medical Sciences, Tehran, Iran; ^2^Department of Pathology and Laboratory Medicine, University of California-Los Angeles (UCLA), Los Angeles, California, USA

**Keywords:** Transcatheter Closure, Secundum Atrial Septal Defect, Chest pain, Patent Foramen Ovale, Complications, Transesophageal Echocardiography

## Abstract

***Introduction:*** We evaluated chest pain alongside other midterm subjective and objective complications of the transcatheter closure of atrial septal defects (ASDs) and patent foramen ovales (PFOs) with various closure devices.

***Methods:*** This cross-sectional study, performed from March 2010 to October 2015 in Rajaie Cardiovascular, Medical, and Research Center, evaluated 313 patients (mean age = 29.12 ± 10 years, 32.9% male) for probable complications associated with the transcatheter occlusion of secundum ASDs (n = 289, mean age = 30.5 ± 11.4 years, 28% male) or PFOs (n = 24, mean age = 42.8 ± 10.2 years). ASD closure was performed under sedation and transesophageal echocardiography (TEE) guidance. Duration of follow-up was 12 ± 3 months (mean follow-up = 11.52 months).

***Results:*** Among the subjective complications, chest pain was the most frequent complaint during the follow-up period and although it was common (7.3%), a clear cardiac etiology was rare. Thirteen (4.2%) patients reported palpitation during the follow-up period, and 4 had documented arrhythmias—including atrial flutter, atrial fibrillation, and 2:1 atrioventricular block. Migraine with or without aura occurred in 1.6% of the patients. Objective complications comprising tamponade, device embolization, and thrombus formation occurred in 6 (1.9%) patients. There was no procedure-related mortality in our patients.

***Conclusion:*** Transcatheter closure of PFOs and secundum-type ASDs in our adult patients using ASD septal occluders was associated with a high degree of success, minimal procedural subjective and objective complication rates, and excellent short- and midterm results. Although chest pain was common after the first month following ASD closure, there was no cardiac death or aortic erosion in 11.52 months follow up.

## Introduction

Interatrial septal defects (ASDs) are congenital heart pathologies and are defined as communications between the 2 atria. These defects comprise patent foramen ovales (PFOs), true defects in the septum primum and secundum, and defects in the sinus venosus and coronary sinus region.^1^

With the introduction of new devices and techniques, transcatheter closure has become the standard procedure for most small- and medium-sized secundum-type ASDs and PFOs in interventional cardiology.^2-4^ In appropriately selected patients with ASDs and PFOs, the effectiveness and safety of transcatheter closure are deemed comparable to those of surgical interventions.^5,6^ ASDs are the most common congenital heart disease in adults; they occur in 1 in every 1500 live births and account for 5% to 10% of all congenital heart diseases.^4,7^ Patients with secundum ASDs are not usually symptomatic; nonetheless, most of them will develop symptoms throughout their lives. The most frequent presentation is exercise intolerance. It is vitally important that the defect be detected early; failure to do so is allied to such serious complications as paradoxical embolism, arrhythmia, pulmonary hypertension, and even right-sided heart failure.^8,9^ Transcatheter ASD closure decreases the right ventricular (RV) volume load, RV size, and pulmonary artery pressure and, thus, confers significant symptomatic improvement.^9^ PFOs affect approximately 25% of the general population.^10^ As an incidental finding in asymptomatic subjects, PFO diagnosis does not necessitate any therapy. On the other hand, PFOs could be a potential risk factor for paradoxical embolism due to right-to-left intracardiac shunts. PFOs have been detected in about 50% of patients with cryptogenic stroke; consequently, a causative relationship between PFOs and/or atrial septal aneurysms and thromboembolic events has been suggested.^11^ In the presence of recurrent transient ischemic attack or stroke, the transcatheter closure of the defect is recommended. However, there is a dearth of well-defined data on the interventional method.^12,13^

For all the studies that have evaluated the objective complications of ASD or PFO closure, the existing literature contains only a few studies on the evaluation of the subjective outcomes. In one of these investigations, Knepp et al^14^ studied 94 patients and reported that 7.4% of the subjects had residual shunts immediately following atrial septal occluder placement. During the follow-up, 4 patients had residual shunts, which were closed for a complete closure rate of 97%. Seven cases developed documented arrhythmias— including supraventricular tachycardia, atrial fibrillation, and premature ventricular beats. There was 1 death in a child due to a cerebral vascular event, 18 months following device placement. Just 1 mild aortic insufficiency was reported.

Accordingly, the present study may be unique insofar as not only did we specifically investigate the subjective complications of the transcatheter closure of ASDs and PFOs, particularly chest pain, but also the age bracket of our patients is relatively older than the age at which such defects are usually diagnosed and treated. (This is due to the specific socioeconomic status of our general population.)

## Materials and Methods

### Patients

The present study enrolled 313 patients who underwent transcatheter closure procedures for secundum ASDs or PFOs at our cardiology department between March 2010 and October 2015. The procedures were performed by experienced attending physicians at Rajaie Cardiovascular, Medical, and Research Center, Tehran, Iran. Of the 313 patients, 284 (90.7%) had secundum-type ASDs and 24 (7.6%) had PFOs that required interventional closure procedures. All the patients were evaluated by transthoracic echocardiography (TTE) and transesophageal echocardiography (TEE) before the procedure. Percutaneous transcatheter closure was performed in the patients with secundum-type ASDs detected by TEE and the presence of significant left-to-right shunting (Qp/Qs >1.5, measured during catheterization), RV dilatation, dyspnea, reduced exercise tolerance, or paradoxical embolism. In the whole study population, pre- and intraprocedural TEE was performed to assess the ASD morphology and adequacy of the defect rims. In the patients with aortic rims smaller than 5 mm, transcatheter closure was performed only if the other rims were adequate. One day after the procedure, TTE was performed to confirm the proper position of the device and the cessation of left-to-right shunting. The patients with PFOs also underwent TTE and TEE before the closure of their defects. After careful evaluation of the PFOs by TEE, closure was performed under TTE guidance.

### Transthoracic and transesophageal echocardiography

All the patients underwent echocardiography by an expert echocardiologist. TTE was performed with a 2.5-MHz transducer in the left decubitus position, and the patients were evaluated through standard imaging windows. TEE was performed with a 7.5-MHz multiplane transducer at 0° (4-chamber view), 45° (aortic short-axis view), and 120° (bicaval view) in mid-esophageal levels for the evaluation of the interatrial septum.

### Follow-up

TTE with intravenous saline contrast injection study was conducted postprocedurally.

### Statistical analysis

The continuous variables are expressed as means ± standard deviations (SDs) and the categorical variables as percentages. The statistical analyses were carried out using SPSS statistical software (version 22, SPSS).

## Results

### Patients

Between March 2010 and October 2015, totally 313 patients (mean age = 29.12 ± 10 y, 32.9% male) underwent the transcatheter closure of PFOs (n = 24, mean age = 42.8 ± 10.2 years, 80% male) and ASDs (n = 289, mean age = 30.5 ± 11.4 years, 28% male) with various devices in our department. In the PFO group, 10 (41.6%) patients had ischemic stroke and 14 (58.3%) had transient ischemic attack. In the patients with PFO closure, the mean diameter of the device was 23.4 ± 3.2 mm (18–30 mm). In the ASD patients, the defect diameter was assessed by TEE before and during the procedure. The mean ASD diameter was 16 ± 4.6 mm, whereas the mean waist diameter of the ASD device was 18.3 ± 5.2 mm (8–30 mm). The baseline characteristics of the study group are depicted in Table 1 and Table 2.

**Table 1:** Patients’ demographic details (N = 313)

**Parameters**	**All Patients (N=313)**	**ASD (n=289)**	**PFO ** **(n=24)**
Age (y)	29.12±10	30.5±11.4	42.8±10.2
Gender, male (%)	103 (32.9%)	81 (28%)	19 (80%)
Defect diameter* (mm)	-	16±4.6	-
Device diameter (mm)	21.1±6.2	18.3±5.2	23.4±3.2

ASD, Atrial septal defect; PFO, Patent foramen ovale *Diameter of the defect was measured by transesophageal echocardiography. The mean diameter was moderate to large. The closure of the larger defects with sufficient rims detected via transesophageal echocardiography needed an expert team, which was not available in that period.

**Table 2:** Periprocedural complications and follow-up data of the study population

	All Patients (N=313)
**Periprocedural complications**	
Thrombosis	0.3%
Tamponade	0.7
Device embolization	1
Erosion event	0
Cardiac death	0
**Follow-up results**	
Chest pain	7.3%
Palpitation	4.2
New arrhythmia	1.3
Migraine with/without aura	1.6
Thromboembolic events	0
Recurrent transient ischemic attack	0
Recurrent ischemic stroke	0

**Figure 1:**
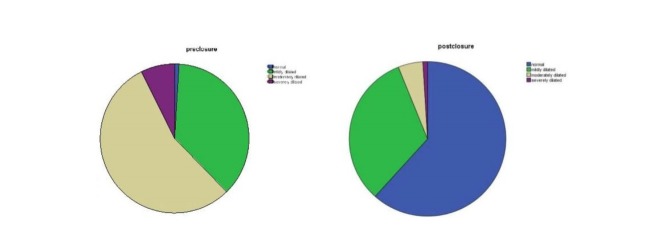
Right ventricular (RV) dilatation: comparison of pre-ASD closure RV size (left) and post-closure RV size at the latest follow-up (right).

The RV size, measured quantitatively by TTE, was recorded prior to closure in all 313 patients. The results showed that 0.9% (n = 3) of the patients had a normal RV size, 36.7% (n = 115) had a mildly dilated RV, 54.9% (n = 172) had a moderately dilated RV, and 7.3% (n = 23) had a severely dilated RV. At the last follow-up (mean follow-up = 11.88), RV size assessment was done in all the patients and revealed that the RV size had returned to normal in 61.6% (n = 193), while 32.2% (n = 101) had persistently mild dilation of the RV, 5.1% (n = 16) had a moderately dilated RV, and 0.9% (n = 3) still had a severely dilated RV (Figure 1). Most of the size resolution in the RV occurred within the first to sixth post-closure months. (The mean percent size reduction was 12.28%, and the mean follow-up time was 3.56 months.)

### Periprocedural objective and subjective complications

#### Subjective complications

Most patients had dyspnea on exertion and palpitation before the procedure; however, new-onset chest pain was the goal of our study. Twenty-three patients reported having chest pain, mostly atypical form, during the follow-up; none of these cases was reported in the first month. All the patients with chest pain received workup; only 2 of them had cardiac causes: myocarditis in 1 case and pericarditis with pericardial effusion with an unknown etiology in the other patient. Classically, patients with myocarditis tend to present with chest pain associated with ECG changes and ST-segment elevation in conjunction with segmental or global wall motion abnormalities on echocardiography. All the patients had ECG and echocardiography in their follow-up, and only 1 patient was diagnosed to have myopericarditis with increased troponin and mild left ventricular dysfunction on TTE. Angiography showed normal coronary arteries.

Thirteen patients reported to have palpitation during the follow-up period: 4 patients in the first month and the rest of them in later visits. However, only 4 patients had documented new-onset arrhythmia. Three of them had supraventricular tachycardia (2 cases of atrial fibrillation and 1 case of atrial flutter) 1 day and 1 month after uncomplicated procedures, respectively; they underwent successful cardioversion. One patient with a large ASD (26 mm) presented to the emergency department with complaints of palpitation and dizziness with 2:1 atrioventricular block on the ECG without any reversible causes 2 weeks after the procedure (device size = 28); consequently, a permanent pacemaker was implanted for him.

New-onset migraine with aura was reported in 3 patients after ASD closure during the follow-up period; migraine with aura was diagnosed with headache and sudden and transient loss of vision unilaterally upon presentation. Additionally, brain magnetic resonance imaging and TEE excluded any possible thromboembolic etiologies 1 month after uncomplicated procedures. Interestingly, new-onset migraine without aura was seen in 2 patients after PFO closure during the follow-up period: 1 of these patients had migraine with concomitant atypical chest pain of no cardiac origin. All 5 patients had improvement by conservative management during the follow-up.

#### Objective complications

Small residual shunts were observed in the first month after ASD closure in 33 patients; nevertheless, at 1 month’s follow-up, there was no residual shunt. There were 6 major procedure-related complications: 2 cases of device embolization to the left ventricle, 1 case of device embolization to the left atrium, 1 case of thrombus formation on the device, and 2 cases of tamponade. The embolized devices were retrieved via open surgery, during which the ASD was closed. One female patient presented to the emergency department complaining of dyspnea on exertion after an uncomplicated ASD closure; she received dual antiplatelet therapy with ASA and Plavix. Nonetheless, after 2 weeks, she complained of dyspnea on exertion (functional class II–III) and echocardiography showed thrombosis on the right-sided disc. Anticoagulation was administered to her, and follow-up echocardiography confirmed a resolved thrombosis. Two patients had small-to-moderate-sized pericardial effusion 1 day and 1 month after uncomplicated procedures with no other complications; they were treated with conservative management. Complications such as tamponade, pericardial effusion, and arrhythmia were excluded in the evaluation of the symptomatic patients postprocedurally.

In the current study, no cases of cardiac erosion, thromboembolic event, recurrent transient ischemic attack, and ischemic stroke were reported (Table 2).

## Discussion

In most centers, the transcatheter closure of ASDs and PFOs has become the method of choice and has, as such, superseded surgical closure. In this report, we presented our experience regarding the transcatheter closure of ASDs and PFOs in a large number of patients. Some advantages of the percutaneous closure of interatrial septal defects include the absence of thoracotomy scar, no need for intensive care unit admission, earlier hospital discharge, lesser blood transfusion, fewer atrial arrhythmias due to the absence of myocardial scar, milder pain, and fewer psychological complications for the patients.^15,16^ There are low mortality and morbidity rates associated with the transcatheter technique. In our opinion, a careful device size selection matched with a stretched ASD diameter is of vital importance. An oversized device can distort the retention discs and impinge on sensitive structures, whereas an undersized device may result in residual shunting and probable early or even late embolization of the device. Thus, TEE monitoring before and after the procedure to confirm the precise placement of the device is critical.

Despite the fact that chest pain was frequent during the follow-up period among our study population, a cardiac etiology was infrequent. Remarkably, there was no chest pain complaint during the first month after atrial septal occluder placement. Very rarely this common and alarming symptom and its causes have been studied in previous reports. In one of those rare investigations, Knepp et al^14^ studied 94 patients and revealed that 18 (19%) patients reported chest pain during the follow-up period. Our study is, therefore, unique in this regard.

Residual shunting is a major concern following a septal occluder positioning. A small residual shunt is a common finding early after ASD closure and it does not seem to lead to long-term complications. Embolization occurred in 3 (1%) of our patients. Device embolization, device thrombosis, and tamponade were the most common major complications. Careful case selection, exact defect-size determination, appropriate device selection, air-embolism prevention, and TEE monitoring during and after the transcatheter procedure can minimize complications. Arrhythmias are the next most common complication. Chiming in with other similar studies, atrial fibrillation was the most frequent arrhythmia but no thromboembolic events occurred in our study.^17^

Palpitation was reported frequently in the post-placement period. No significant migraine headache was developed in our patients. The association between PFOs and migraine headache has been studied in many investigations.^18,19^ Prospective trials have also assessed PFO closure with an implantable device as a treatment option.^20^ Despite the overlap between PFOs and secundum ASDs in adults, the relationship between migraine and the atrial septal occluder closure of secundum ASDs could not be generalized. Overall, the literature supports the concept that both the traditional surgical technique AND the newer transcatheter method in the treatment of secundum ASDs are associated with complications; be that as it may, there is a tendency toward the use of the transcatheter method to reduce the complications. Although the immediate and midterm results of transcatheter interventions in the interatrial septum appear to be desirable in terms of efficacy and safety compared with surgical procedures, data on long-term results are limited.^6^ The most important drawbacks of the devices implanted in the interatrial septum are erosion of the aortic wall and, in particular, aortic perforation caused by the device.^21,22^ Despite the technical possibility of the transcatheter ASD closure in adult cases without adequate aortic rims, there is no sufficient information on the long-term safety of this procedure. The most important factors associated with device-related erosion have been reported to be the implantation of an oversized device and the moving of the device relative to the heart.^22,23^ Transcatheter PFO closure has been reported to be safe and effective in these patients due to the increased risk for paradoxical embolism.^24^ Recent reports have suggested that even in patients with mutations in factor V Leiden, factor X, factor VIII, protein C, protein S, and MTHFR as well as those with positive antiphospholipid/anticardiolipin antibodies, transcatheter PFO closure may be beneficial.^24^

## Study Limitations

There are some limitations to our study. First, this is a case-control study in which the data were collected retrospectively. Second, our findings predominantly reflect the midterm safety and efficacy of the devices; thus, long-term follow-up results would help to clarify data on the safety of these devices. Third, fewer patients underwent PFO closure than ASD closure because the indications for ASD closure are more robust. In contrast the value of PFO closure compare to the anticoagulation in cryptogenic stroke is still under investigation. Fourth, our study presents single-center experience with various devices, while multicenter, randomized, and comparative studies are needed to assess the safety and efficacy of the devices in different patient groups. One of the important factors for the success of transcatheter ASD and PFO closure is the experience of the center.^25^ In this respect, our center is considered a high-volume clinic for interventional procedures involving the interatrial septum. It is deserving of note that strict case selection, proficiency, and careful device selection may have led to very low rates of complications such as device embolization in our center.

## Conclusion

In summary, transcatheter interventions in the interatrial septum for the treatment of moderate-sized secundum-type ASDs are successful and safe, although there may be some subjective and objective complications. Therefore, the application of such devices necessitates careful attention because of the possibility of failure or the embolization of the device. With respect to chest pain (our main variable), our results demonstrated that although it was common after the first month following ASD closure, there was no aortic erosion in moderate-sized ASDs at 11.52 months’ follow-up with these special devices.

## Ethical approval

All the patients’ data were confidential and only the codes number recorded in data collection sheets.

## Competing interests

All authors declare no competing financial interests exist.
